# Development of Fe_3_O_4_@SiO_2_ Magnetic Nanobeads Decorated with Graphene Oxide for Improved DNA Separation

**DOI:** 10.3390/nano16211428

**Published:** 2026-11-05

**Authors:** Camellia Panatarani, Karisa A. Fauzi, Ferry Faizal, Adi Darmawan, Nur L. Hamidah, Eka C. Prima, Mia Miranti, Yolani Syaputri, Bhawana Jain, Sundoro Y. Azhary, I M. Joni

**Affiliations:** 1 Department of Physics, Faculty of Mathematics and Natural Sciences, Universitas Padjadjaran, Jalan Raya Bandung-Sumedang Km 21 Jatinangor, Sumedang 45363, Indonesiaimadejoni@phys.unpad.ac.id (I.M.J.); 2 Functional Nano Powder University Center of Excellence, Universitas Padjadjaran, Jalan Raya Bandung-Sumedang KM 21, Jatinangor, Sumedang 45363, Indonesia; 3 Department of Chemistry, Diponegoro University, Tembalang, Semarang 50275, Indonesia; adidarmawan@live.undip.ac.id; 4 Department of Engineering Physics, Faculty of Industrial Technology and System Engineering, Institut Teknologi Sepuluh Nopember, Surabaya 60111, Indonesia; nurlaila@its.ac.id; 5 Department of Physics, Universitas Pendidikan Indonesia, Jl. Dr. Setiabudhi No. 229, Bandung 40154, Indonesia; ekacahyaprima@upi.edu; 6 Department of Biology, Faculty of Mathematics and Natural Sciences, Universitas Padjadjaran, Jalan Raya Bandung-Sumedang Km 21 Jatinangor, Sumedang 45363, Indonesia; mia.miranti.rustama@unpad.ac.id (M.M.);; 7 Department of Chemical Sciences, Siddhachalam Laboratory, Raipur 493221, Chhattisgarh, India

**Keywords:** magnetic nanobeads, graphene oxide, DNA separation, silica, surface functionalized

## Abstract

In molecular biology, magnetic nanobeads play a key role in the separation and purification of DNA and RNA, which are necessary for procedures such as PCR. However, conventional magnetic nanobeads suffer from instability, aggregation, and poor surface functionality. The objective of this study was to synthesize and characterize graphene oxide (GO) -conjugated Fe_3_O_4_@SiO_2_ magnetic nanobeads for DNA isolation. The large surface area and functional groups of GO could bring about strong interactions with DNA, thus enhancing the binding and separation efficiency. Here, Fe_3_O_4_ nanoparticles are chosen as the core material because of the high magnetic saturation and biocompatibility. The silica shell can improve stability and serve as a platform for further functionalization. Amino functionalization of Fe_3_O_4_@SiO_2_ allows conjugation of GO at reactive sites. The stability and functionalization of the nanobeads were confirmed by Fourier transform infrared spectroscopy (FTIR), X-ray diffraction (XRD), X-ray fluorescence (XRF), particle size distribution, zeta potential, and electrophoretic mobility. The optimal GO to Fe_3_O_4_@SiO_2_-NH_2_ conjugation ratio (5:1) balanced adequate GO coverage and controllable particle dimension. The increased negative charge improved colloidal stability and reduced aggregation. The enhanced electrophoretic mobility ensures efficient separation of DNA. These findings highlight the potential of GO-conjugated Fe_3_O_4_@SiO_2_ magnetic nanobeads as promising tools for advanced DNA separation techniques.

## 1. Introduction

Magnetic nanobeads represent a versatile platform for nucleic acid extraction and purification, offering rapid separation, high recovery efficiency, and ease of operation. Their use in the isolation of DNA and RNA plays a vital role in molecular biology applications such as polymerase chain reaction (PCR), gene expression studies, and genomic analyses. Their usefulness comes from the fact that magnetic nanobeads can be controlled with magnetic fields, which enable the recovery of biomolecules in an accurate and effective manner [1]. Traditional magnetic beads usually suffer from agglomeration, instability, and restricted surface functionality [2]. This points out the need for developing advanced material designs that improve surface properties without sacrificing structural integrity [3]. Since it directly affects the binding efficiency and purity of the extracted DNA, the relationship between the electrophoretic mobility of magnetic beads and DNA extraction efficiency is crucial in this situation. Electrophoretic mobility is essential in optimizing extraction since it dictates how well magnetic beads can interact with DNA [4]. The surface characteristics of magnetic beads, including their charge and size significantly influence their electrophoretic mobility, directly affecting their ability to bind DNA [5]. Moreover, the adjustment in the environmental parameters such as pH and ionic strength further enhanced the DNA binding to magnetic beads, resulting in improved yields in extraction [6,7]. These results emphasize that bead characteristics and reaction conditions must be optimized to get the best outcomes from DNA extraction procedures.

The functionalization of magnetic nanobeads with graphene oxide (GO) is a promising development in this area. Due to its aromatic nature, which promotes strong π-π stacking with DNA bases and increases adsorption efficiency, GO has an improved binding mechanism [8]. Furthermore, functional groups such as -COOH and -OH improve the DNA binding interaction sites, further improving extraction efficiency [9]. Moreover, magnetic/GO nanocomposites have proven to be very successful in the isolation of DNA and were able to maintain this capability over several extraction cycles [10]. These findings show that GO-functionalized magnetic beads can assist in efficient DNA extraction. However, the use of GO in magnetic nanobeads for DNA separation remains unexplored despite its potential, especially when it comes to maximizing its conjugation with silica-coated magnetic cores.

Due to their high magnetic saturation, biocompatibility, and easy modification, magnetic Fe_3_O_4_ nanobeads are ideal as a core material. Nevertheless, oxidation causes their magnetic properties to decrease over time. Covering the Fe_3_O_4_ core with a silicon dioxide shell (SiO_2_) reduces the limitation. This creates a strong core–shell structure that protects the magnetic core from oxidation and provides a stable, chemically inert surface for additional functionalization [11]. The SiO_2_ layer provides more stability to the nanobeads, improves their surface, and facilitates adsorption for various applications [12]. Indeed, the SiO_2_ coating significantly improves the stability of the nanobeads, enlarges the surface area, and enhances adsorption, hence making them versatile. Additionally, this silica coating provides a strong and chemically inert base for additional functionalizations, facilitating the creation of features specifically suited to specific applications.

A well-known functionalization technique uses silane coupling agents such as 3-aminopropyltriethoxysilane (3-APTES) to add amino groups (-NH_2_) onto the surface of Fe_3_O_4_@SiO_2_ nanobeads [13]. N-Hydroxysuccinimide (NHS) and 1-(3-dimethylaminopropyl)-3-ethylcarbodiimide hydrochloride (EDC-HCl) are chemical coupling agents that allow these amino groups to form reactive sites necessary for covalent interaction with graphene oxide (GO) [14]. This stepwise modification process, starting with silica coating and ending with aminofunctionalization, guarantees that the magnetic nanobeads retain their structural integrity while maintaining the functional surfaces required for efficient GO conjugation.

When GO is conjugated to Fe_3_O_4_@SiO_2_-NH_2_, a hybrid material is created with improved surface functionality and stability. GO expands the surface available for biomolecular interactions and adds more functional groups. However, obtaining the ideal GO to Fe_3_O_4_@SiO_2_-NH_2_ conjugation ratio is crucial. While insufficient coverage limits functional performance, excessive GO loading can lead to particle aggregation and reduced colloidal stability. This balance must be found to maximize the material’s effectiveness in DNA separation applications.

Therefore, this work aims to investigate the effectiveness of various surface-functionalized magnetic nanobeads, particularly Fe_3_O_4_@SiO_2_ and its graphene oxide conjugates, in improving the yield and purity of the genomic DNA extraction process. To investigate the effect of GO-conjugated on the Fe_3_O_4_@SiO_2_ in the DNA separation, this magnetic material was systematically characterized. X-ray diffraction (XRD) and X-ray fluorescence (XRF) were used to verify the structural stability of the Fe_3_O_4_ core and alterations. The gradual alterations, such as silica coating, amino functionalization, and GO conjugation, were confirmed by Fourier-transform infrared spectroscopy (FTIR). The physical and electrostatic stability of the nanobeads was determined by zeta potential, particle size analysis (PSA), and electrophoretic mobility tests. The performance of GO conjugation on the DNA separation was investigated using nanodrop characterization.

## 2. Materials and Methods

### 2.1. Materials

The materials utilized to synthesize Fe_3_O_4_@SiO_2_-NH_2_ nanobeads and graphene oxide (GO) were sourced from commercial suppliers. Graphite powder (Merck, Jakarta, Indonesia CAS-No: 7782-42-5) was the main precursor used to prepare graphene oxide. Hydrochloric acid (HCl, Merck, Jakarta, Indonesia CAS-No: 7647-01-0), sulfuric acid (H_2_SO_4_, Merck, Jakarta, Indonesia CAS-No: 7664-93-9), potassium permanganate (KMnO_4_, Merck, Jakarta, Indonesia CAS-No: 7722-64-7), hydrogen peroxide (H_2_O_2_, Merck, Jakarta, Indonesia CAS-No: 7722-84-1), and sodium nitrate (NaNO_3_, PT. Brataco) were also used. The silicone oil, table salt, ethanol, Iron(II) chloride tetrahydrate (FeCl_2_·4H_2_O, Merck, Jakarta, Indonesia CAS-No: 13478-10-9) and iron(III) chloride hexahydrate (FeCl_3_·6H_2_O, Merck, Jakarta, Indonesia CAS-No: 10025-77-1) were utilized as iron precursors, and 1.5 M ammonia solution (Merck, Jakarta, Indonesia CAS-No: 1336-21-6) as a precipitating agent. For the silica (SiO_2_) shell, tetraethyl orthosilicate (TEOS, Merck, Jakarta, Indonesia CAS-No: 78-10-4) was used. Nitrogen gas was used to maintain an inert atmosphere during synthesis, while polyethylene glycol (PEG 6000, Merck, Jakarta, Indonesia CAS-No: 25322-68-3) acted as a steric stabilizer. PEG 6000 prevented Fe_3_O_4_ nanoparticle aggregation by providing steric hindrance, thereby improving colloidal stability in aqueous suspension.

3-aminopropyltriethoxysilane (3-APTES, ≥98.0%, Merck, Jakarta, Indonesia CAS-No: 919-30-2) was used as the silane coupling agent for amino functionalization of Fe_3_O_4_@SiO_2_ nanobeads. N-hydroxysuccinimide (NHS, ≥97.0%, Merck, Jakarta, Indonesia CAS-No: 56480-100) and 1-(3-dimethylaminopropyl)-3-ethylcarbodiimide hydrochloride (EDC·HCl, ≥98.0%, Merck, Jakarta, Indonesia CAS-No: 25952-53-8) were used as chemical coupling agents for covalent bonding in the further functionalization of the compound. Deionized water was used in all reactions performed during synthesis.

### 2.2. Methods

#### 2.2.1. Synthesis of Graphene Oxide (GO)

A modified Hummers’ method, was used to produce graphene oxide (GO) from graphite [15]. In an Erlenmeyer flask, 2 g of graphite powder and 1 g of sodium nitrate were mixed with 45 milliliters of sulfuric acid and swirled and kept in an ice bath (0–5 °C) for half an hour. Next, to keep the mixture’s temperature below 20 °C, 5 g of potassium permanganate (KMnO_4_) was prepared and gradually added, requiring caution throughout the addition process. After stirring the mixture for another half hour, the ice bath was replaced with a silicone oil bath and the mixture was kept at 35 °C for one hour with constant stirring. Next, 45 mL of distilled water was gradually added to the reaction mixture to dilute it, raising the temperature to 98 °C with continuous stirring for 40 min. A further 200 mL of distilled water and 25 mL of hydrogen peroxide (H_2_O_2_) were added, and the mixture was stirred for another half hour followed by washing with deionized water. Lastly, the sample was purified using 10 milliliters of hydrochloric acid (HCl) solution in deionized water, the precipitate was separated and the sample was sonicated to get exfoliated graphene oxide. The final GO solution was dried at 90 °C.

#### 2.2.2. Synthesis of Fe_3_O_4_ and Fe_3_O_4_@SiO_2_

Fe_3_O_4_@SiO_2_ was synthesized using the procedure outlined in the previous study [16,17]. To prepare the magnetite (Fe_3_O_4_) core, 1.99 g of FeCl_2_·4H_2_O and 5.41 g of FeCl_3_·6H_2_O were dissolved in 50 milliliters of deionized (DI) water with continuous stirring at 500 rpm for 15 min. To this solution, 1.15 g of PEG 6000 was added and stirred for 15 min. Then, this solution was added dropwise using a micropump to another beaker containing 1.15 g of PEG 6000 with 50 milliliters of 1 M ammonia solution while being stirred in the same manner and the mixture was agitated at 500 rpm. Nitrogen gas (N_2_) was added to the mixing environment after the dropwise process was finished, and the stirring speed was raised to 700 rpm for an hour. Afterward, the stirring speed was gradually reduced to 500 rpm, followed by centrifugation and washing to obtain the sample particles. The fine particles were dispersed as the suspension medium in ethanol or DI water.

To enhance DNA binding performance, the magnetic core particles were functionalized with silica through adding 5 mL Fe_3_O_4_ solution to 2.3 mL of tetraethyl orthosilicate (TEOS) under stirring at 500 rpm for 15 min. During mixing, 0.35 mL of 1.5 M ammonia solution diluted with 0.7 mL DI water was introduced into the Fe_3_O_4_-TEOS mixture using a micropump while maintaining the stirring speed at 500 rpm. This process yielded a milky-white suspension. The silica-coated suspension was separated from the primary silica solution using a magnetic bar around the tube to isolate the functionalized particles.

#### 2.2.3. Surface Functionalization of Fe_3_O_4_@SiO_2_

The Fe_3_O_4_@SiO_2_ particles were functionalized with 3-APTES [18]. Twenty milliliters of toluene were mixed with one gram of Fe_3_O_4_@SiO_2_ particles, and the mixture was vigorously agitated for half an hour. Two milliliters of 3-APTES were then added to the mixture. The reaction mixture was heated to 120 °C and refluxed for five hours while continuously stirred in a nitrogen environment. The sample was collected using a magnet once it had cooled to room temperature and cleaned adequately with ethanol and toluene. After that, the functionalized particles were dried for 12 h at 60 °C and 0.05 MPa of pressure in a vacuum oven.

#### 2.2.4. Conjugation of GO onto Fe_3_O_4_@SiO_2_ Surface

Graphene oxide (GO) was conjugated onto the surface of Fe_3_O_4_@SiO_2_ using N-hydroxysuccinimide (NHS) and 1-(3-dimethylaminopropyl)-3-ethylcarbodiimide hydrochloride (EDC-HCl) [19]. GO (50 mg, 250 mg, and 375 mg) was dispersed in 100 mL of deionized (DI) water and sonicated for 30 min to ensure uniform dispersion. Subsequently, 16 mg of NHS and 20 mg of EDC-HCl were added to the GO suspension at concentrations of 0.5 mg/mL, 2.5 mg/mL, and 3.75 mg/mL. Fifty milligrams of Fe_3_O_4_@SiO_2_ particles were introduced into the mixture, followed by mechanical stirring at 80 °C for 2 h. After cooling to room temperature, the sample was collected by centrifugation to obtain the precipitate. The precipitate was washed multiple times using DI water until the pH reached six and was then re-dispersed in 100 mL of DI water.

#### 2.2.5. Genomic DNA Extraction

**Figure 1:**
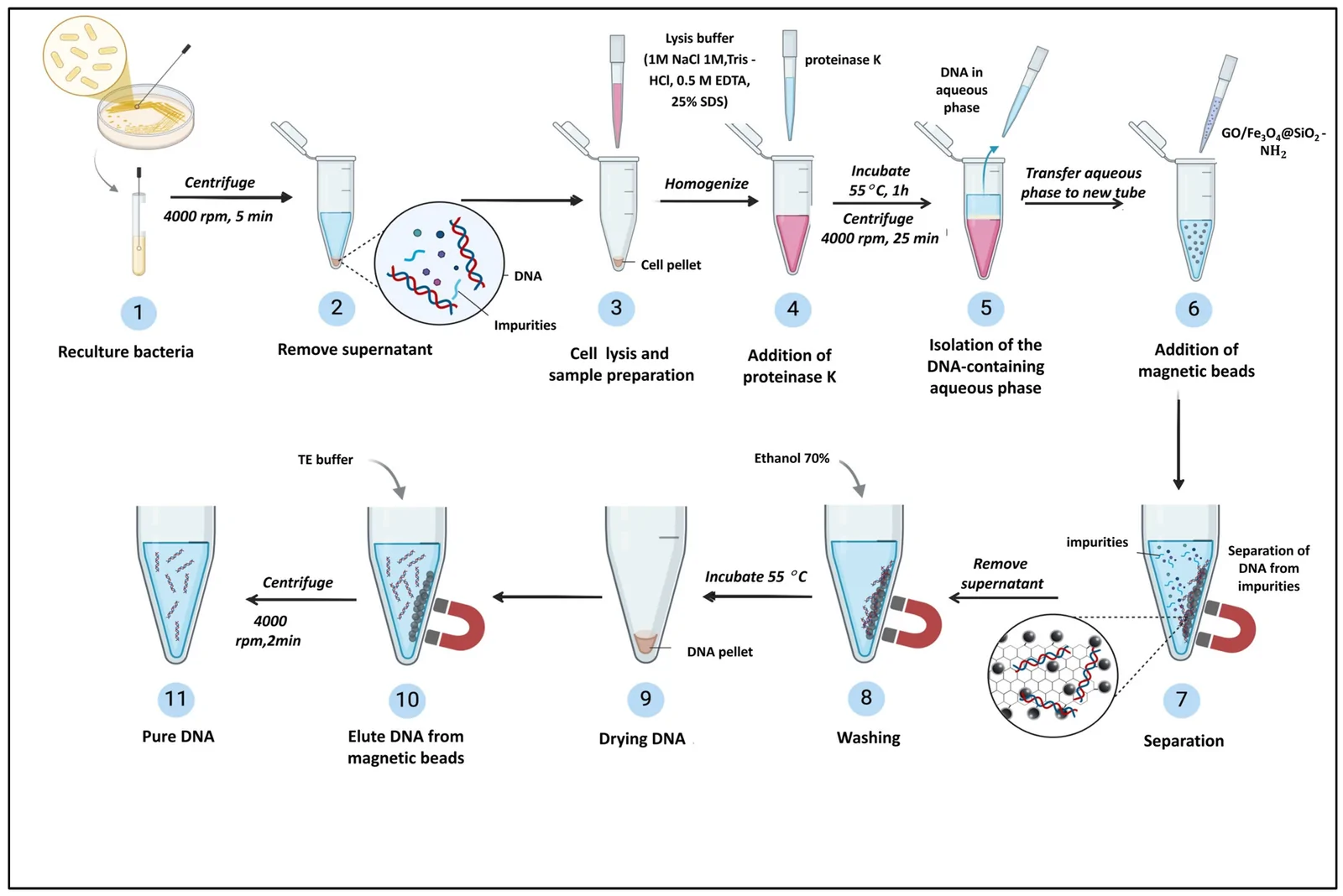
Schematic diagram of genomic DNA extraction.

Schematic diagram of genomic DNA extraction using magnetic nanobeads as shown in Figure 1. The extraction of genomic DNA of bacteria (*Kosakonia cowanii*) by magnetic nanobeads was carried out as follows: a total of four DNA isolation samples were conducted with different variations of magnetic nanobeads. The bacteria were grown on nutrient broth (NB) and incubated at room temperature for 16–24 h. Each sample was worked with as follows: One mL of bacterial culture in the NB medium was centrifuged at 4000 rpm for 5 min. This stage was carried out three times until the total mass of bacteria obtained was from a total volume of 3 mL NB. The obtained pellet was added to 600 µL of lysis buffer (1 M NaCl, 1 M Tris–HCl, 0.5 M ethylenediaminetetraacetic acid (EDTA), and 25% SDS). An additional 3 µL of proteinase K (10 mg/mL) was added and incubated at 55 °C for 1 h in a water bath. During the incubation, the sample was gently shaken every 5 min. Following centrifugation at 4000 rpm for 25 min at room temperature, the supernatant was transferred to a new micro-tube. In order to purify genomic DNA, 200 μL of binding buffer (1.25 mM NaCl and 80% PEG [*w*/*v*]) and 0.4 mg magnetic nanobeads per milliliter of binding buffer were added to the supernatant. Afterward, the samples were incubated at room temperature for 15 min. Then, the particles were separated with a magnetic rack and the supernatant was discarded. The pellet was washed twice with 200 µL of 70% ethanol and dried at room temperature. The pellet was added to 100 µL TE buffer (10 mM Tris–HCl, 1 mM EDTA, and pH 8.0), and incubated at 55 °C for 15 min with gentle shaking to elute genomic DNA. Following centrifugation at 4000 rpm for 2 min and using a magnetic rack, the genomic DNA supernatant was transferred to a new microtube and stored at −20 °C. Four formulations were evaluated: Fe_3_O_4_@SiO_2_, GO/Fe_3_O_4_@SiO_2_-NH_2_ (1:1), GO/Fe_3_O_4_@SiO_2_-NH_2_ (5:1), and GO/Fe_3_O_4_@SiO_2_-NH_2_ (7.5:1). Fe_3_O_4_@SiO_2_ was used as the non-GO reference material, whereas Fe_3_O_4_@SiO_2_-NH_2_ served as an intermediate for GO conjugation and was therefore not evaluated as a separate genomic DNA extraction formulation.

### 2.3. Characterization

Several characterization techniques were performed on the synthesized and modified materials to assess their structural, chemical, morphological, and functional characteristics. X-ray diffraction (XRD) analysis was carried out using a D8 Advance system (Bruker) with Cu Kα radiation (λ = 1.5406 Å) to examine the crystalline structure and identify phase changes in the Fe_3_O_4_ magnetic core. The average crystallite size of Fe_3_O_4_ was estimated using the Scherrer equation, (*D* = *Kλ*/(*βcos* (*θ*)), where (*D*) is the crystallite size, (*K*) is the shape factor (0.9), (*λ*) is the X-ray wavelength, (*β*) is the full width at half maximum (FWHM) of the selected diffraction peak in radians, and (*θ*) is the corresponding Bragg angle. X-ray fluorescence (XRF) (Nex CG XRF Analyzer— Applied Rigaku Technologies, Cedar Park, Texas, USA).) was used to analyze the chemical composition to verify that the silica coating was successful. The materials’ surface functional groups were identified using Fourier-transform infrared spectroscopy (FTIR) (Alpha II spectrometer—Bruker Optik GmbH, Ettlingen, Germany).

For DLS-based particle-size and zeta-potential measurements, the samples were dispersed at a concentration of 5 mg in 10 mL of distilled water, with the dispersion pH maintained at approximately 6–7. Zeta-potential measurements were performed using a HORIBA SZ-100, Kyoto, Japan, nanoparticle analyzer. Electrophoretic mobility was measured by laser Doppler electrophoresis, and the zeta potential was calculated from the measured electrophoretic mobility using the Smoluchowski model. The particle sizes obtained by DLS are reported as hydrodynamic diameters and therefore represent the dimensions of the dispersed particles and/or particle assemblies in suspension rather than the primary-particle size observed by electron microscopy. Since zeta potential is dependent on the dispersion medium and pH, the reported values are interpreted specifically under these measurement conditions. Scanning electron microscopy (SEM) imaging was conducted using the SU3500 Imaging SEM (Hitachi High-Technologies Corporation, Tokyo, Japan) to observe the Fe_3_O_4_ and GO morphology as a core material for fabricating functionalized magnetic nanobeads. We have also used HRTEM 9500 (Hitachi, Kyoto, Japan,) with LaB6 as a source of electrons at 300 kV to study the size and morphology of the nanoparticle samples and their compositions. The magnetic properties were measured by a vibrating sample magnetometer (VSM) (ADE, EV-7VSM, Lowell, MA, USA).

## 3. Results and Discussion

### 3.1. Structural and Compositional Analysis

**Figure 2:**
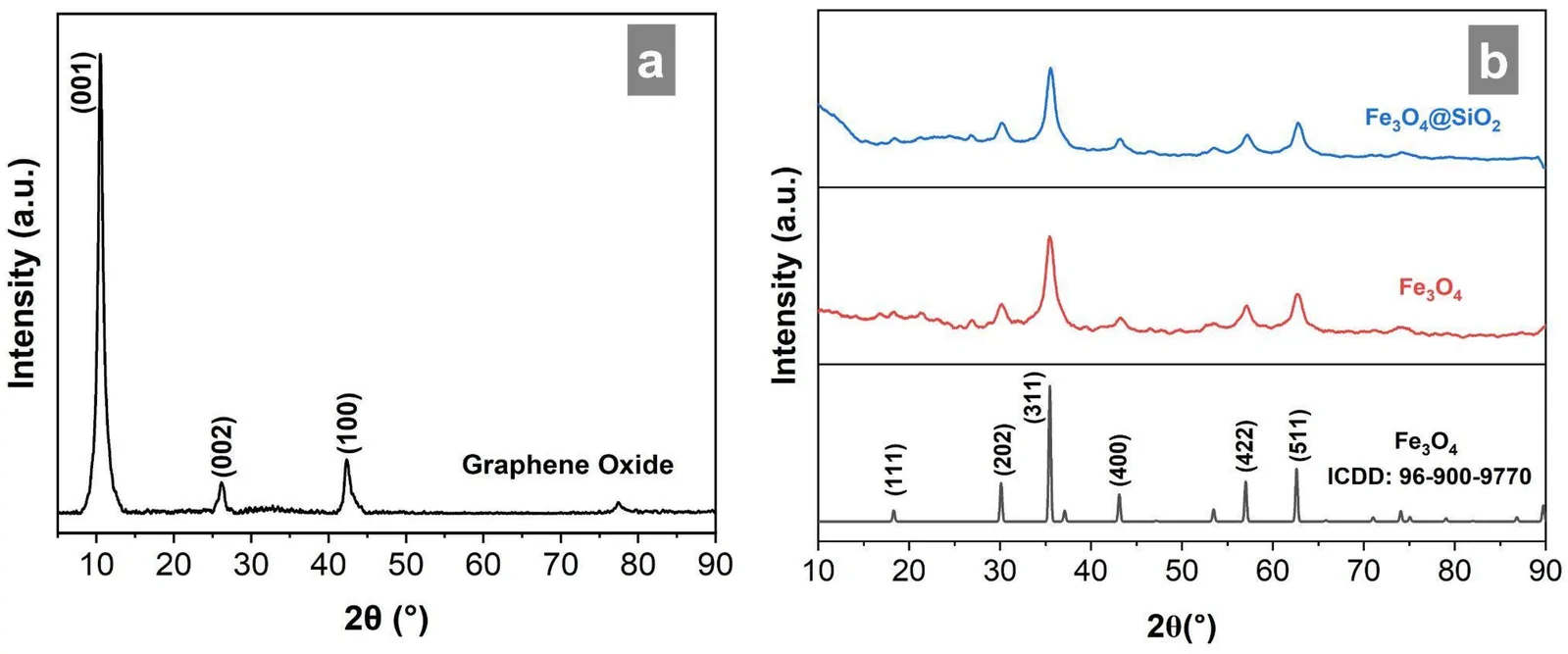
XRD patterns of (**a**) graphene oxide (GO), (**b**) Fe_3_O_4_ and Fe_3_O_4_@SiO_2_.

X-ray diffraction (XRD) was used to examine the crystalline structures of graphene oxide (GO), magnetite nanobeads (Fe_3_O_4_), and silica-coated magnetite (Fe_3_O_4_@SiO_2_). XRD patterns of graphene oxide (GO), Fe_3_O_4_, and Fe_3_O_4_@SiO_2_ are as shown in Figure 2. Due to the addition of oxygen-containing functional groups, graphite was successfully oxidized to GO with an enlarged interlayer gap, as seen by the distinctive peak in the XRD pattern of GO (Figure 2a) at about 2θ = 10°, which corresponds to the (001) plane [20]. Specific diffraction peaks for Fe_3_O_4_ nanobeads (Figure 2b) were visible at 2θ ≈ 30.1°, 35.4°, 43.1°, 57.0°, and 62.6°, corresponding to the (220), (311), (400), (511), and (440) planes, respectively [21]. These peaks confirm the formation of Fe_3_O_4_ nanobeads with the typical cubic spinel structure of magnetite. Using the Scherrer equation applied to the (311) peak, the average crystallite size of Fe_3_O_4_ nanoparticles was estimated to be ~10 nm. After silica coating to form Fe_3_O_4_@SiO_2_, the main Fe_3_O_4_ diffraction peaks remained essentially unchanged, indicating that the silica coating did not alter the crystalline structure of the Fe_3_O_4_ core. In Figure 2b, the broad peak near 2θ ≈ 20°, characteristic of amorphous silica, is not clearly detectable. This is attributed to the thin SiO_2_ layer, which produces a low-intensity amorphous signal that may overlap with the background noise.

The elemental composition of the Fe_3_O_4_ and Fe_3_O_4_@SiO_2_ samples was evaluated using X-ray fluorescence (XRF) analysis. Fe was the dominant element in the Fe_3_O_4_ sample, while the Fe_3_O_4_@SiO_2_ sample showed a marked increase in Si content accompanied by a decrease in the relative Fe content, supporting the successful incorporation of the silica coating. The detailed elemental composition is provided in Appendix A.1 (Table A1).

### 3.2. Surface Morphology and Functional Group Characterization

**Figure 3:**
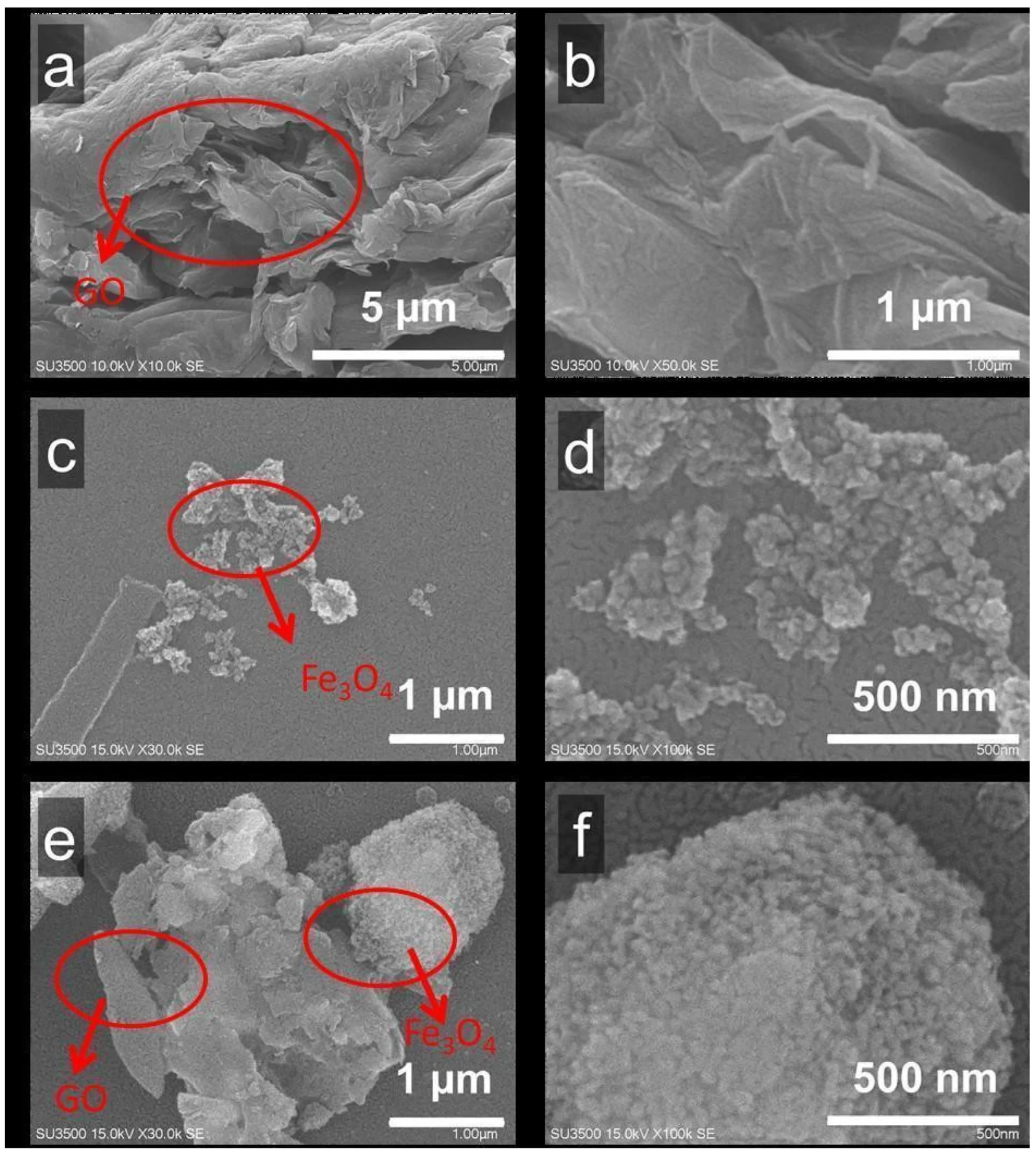
SEM images with varying magnifications of the GO sample (**a**,**b**), Fe_3_O_4_ sample (**c**,**d**), and Fe_3_O_4_@SiO_2_ sample (**e**,**f**).

Figure 3 displays SEM images of graphene oxide (GO) sheets, with (a,b) a clear flaky structure showing exfoliated GO has been synthesized, Fe_3_O_4_ nanobeads (c,d), and Fe_3_O_4_@SiO_2_ nanobeads (e,f) at various magnifications, providing detailed information on their morphologies. The morphology of the GO sample is shown in Figure 3a,b, which is distinguished by its layered and wrinkled sheet-like structure. Under increased magnification, the surface appears wrinkled and corrugated, which is characteristic of graphene oxide [22]. The oxidation process that affects the oxygen-containing functional groups and crystallinity gives GO its distinctive appearance when it is produced using techniques such as the modified Hummers’ method [23]. These characteristics give graphene oxide (GO) an exceptionally high surface area, which makes it perfect for biomolecular interactions and the production of hybrid materials.

The Fe_3_O_4_ particles in Figure 3c have a fairly consistent morphology with an approximately spherical to polygonal shape. Fe_3_O_4_ particles with nanoscale morphology were shown to have been successfully prepared when viewed at higher magnification in Figure 3d, which is beneficial for integration into composite materials. An SEM observation of Fe_3_O_4_@SiO_2_ is shown in Figure 3e,f. The particle clusters consist of nanoscale Fe_3_O_4_@SiO_2_ primary particles when comparing the results of observations at 100,000× magnification with those at 30,000× magnification. The SiO_2_ coating on the Fe_3_O_4_ particles enables the formation of more homogeneous primary particles than Fe_3_O_4_ particles alone. According to these results, silica-coated magnetic nanobeads increase the surface area and produce a more uniform and smaller particle size, which is favorable for additional functionalization and use as a magnetic nanobeads application.

**Figure 4:**
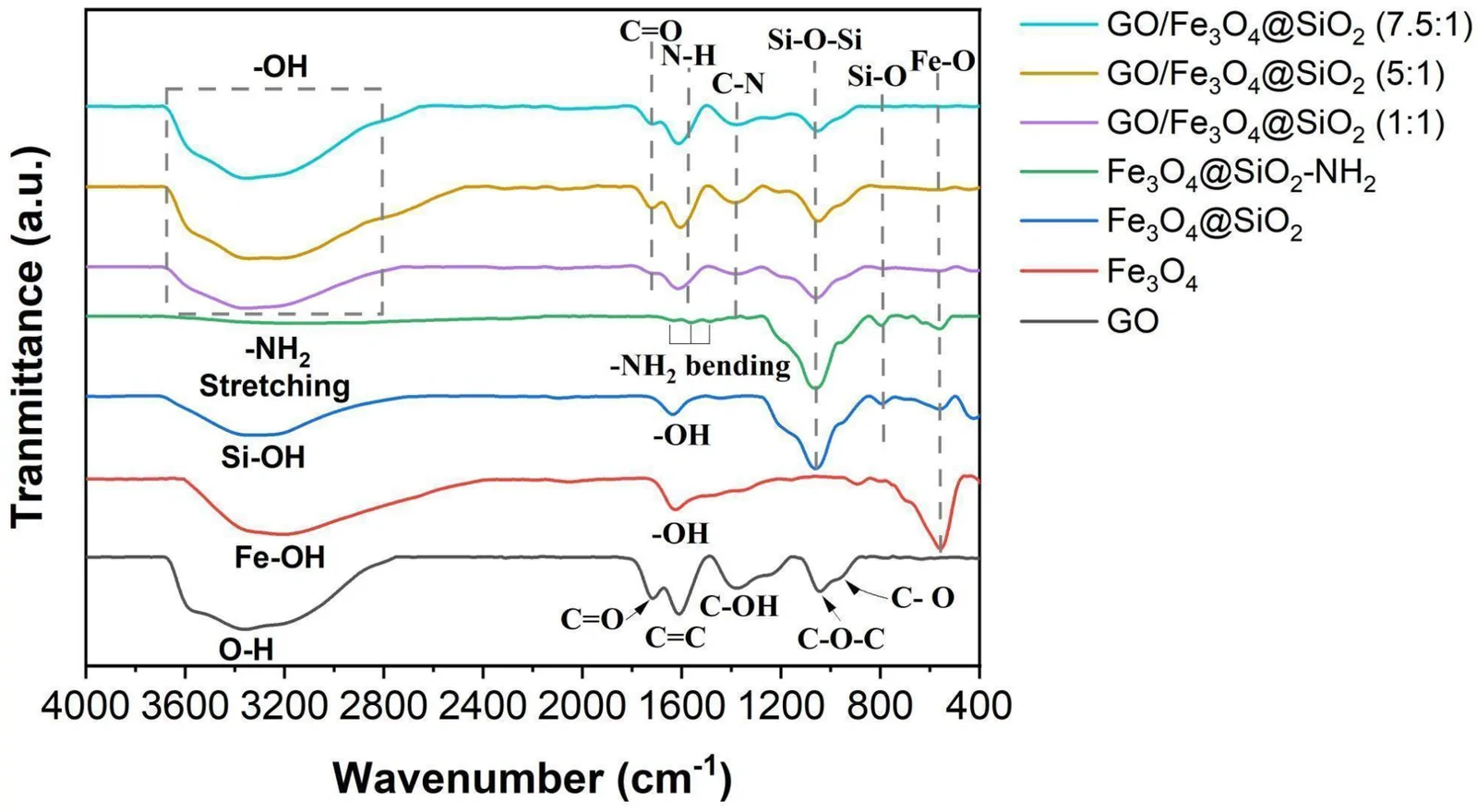
FT-IR characterization results for various samples.

Graphene oxide (GO) is essential to the functionalization of Fe_3_O_4_ magnetic beads for DNA separation due to its unique chemical and structural characteristics. GO’s high surface area, aromatic ring structure, and the presence of oxygen-containing functional groups such as carboxyl (-COOH) and hydroxyl (-OH) provide strong π-π stacking interactions with DNA nitrogenous bases [24]. These interactions enhance the effectiveness of DNA binding and separation. Fourier-transform infrared spectroscopy (FTIR) was used to confirm the functional groups required for these interactions, and the results are displayed in Figure 4. The successful oxidation of graphite to graphene oxide (GO) with numerous oxygen-containing functionalities was validated by the GO spectrum, which showed distinctive absorption bands at about 3420 cm^−1^ (O-H stretching), 1720 cm^−1^ (C=O stretching from carboxyl groups), and 1050 cm^−1^ (C-O stretching) [25].

For Fe_3_O_4_ nanobeads, a prominent absorption band at 580 cm^−1^ was observed, corresponding to the Fe-O vibration, indicating the presence of magnetite. New absorption bands at about 1100 cm^−1^, linked to Si-O-Si stretching vibrations, are shown as peaks after coating with silica to generate Fe_3_O_4_@SiO_2_, indicating the creation of the silica layer [25]. The shielding action caused the Fe-O band to become less intense, further demonstrating the silica layer’s presence.

Functionalization of Fe_3_O_4_@SiO_2_ with amino groups (Fe_3_O_4_@SiO_2_-NH_2_) was confirmed by the appearance of absorption bands at 3420 cm^−1^ and 1630 cm^−1^, corresponding to N-H stretching and bending vibrations, respectively [26]. These amino groups help generate amide linkages, which promote covalent bonding with GO. The FTIR spectra of GO/Fe_3_O_4_@SiO_2_-NH_2_ samples with varying conjugation ratios (1:1, 5:1, 7.5:1) showed successful conjugation. Three distinctive peaks of the stretching vibration of C=O (amide bond Ι at 1651 cm^−1^), the in-plane bending vibration of N-H (amide bond ΙΙ at 1528 cm^−1^), and the stretching vibration of C-N (amide bond ΙΙΙ at 1451 cm^−1^) support these findings [19]. FT-IR spectra showed amide I, II, and III peaks, indicating covalent bonding between GO and amino-functionalized beads. To rule out false positives from physically adsorbed GO, control spectra of physically mixed GO and Fe_3_O_4_@SiO_2_-NH_2_ were recorded. These lacked the characteristic amide peaks, confirming that the observed signals arise from covalent conjugation. The strength of GO-associated bands increased with increasing GO content, thus showing the successful incorporation of GO into the magnetic nanobeads.

### 3.3. Particle Size and Electrokinetic Behavior

**Figure 5:**
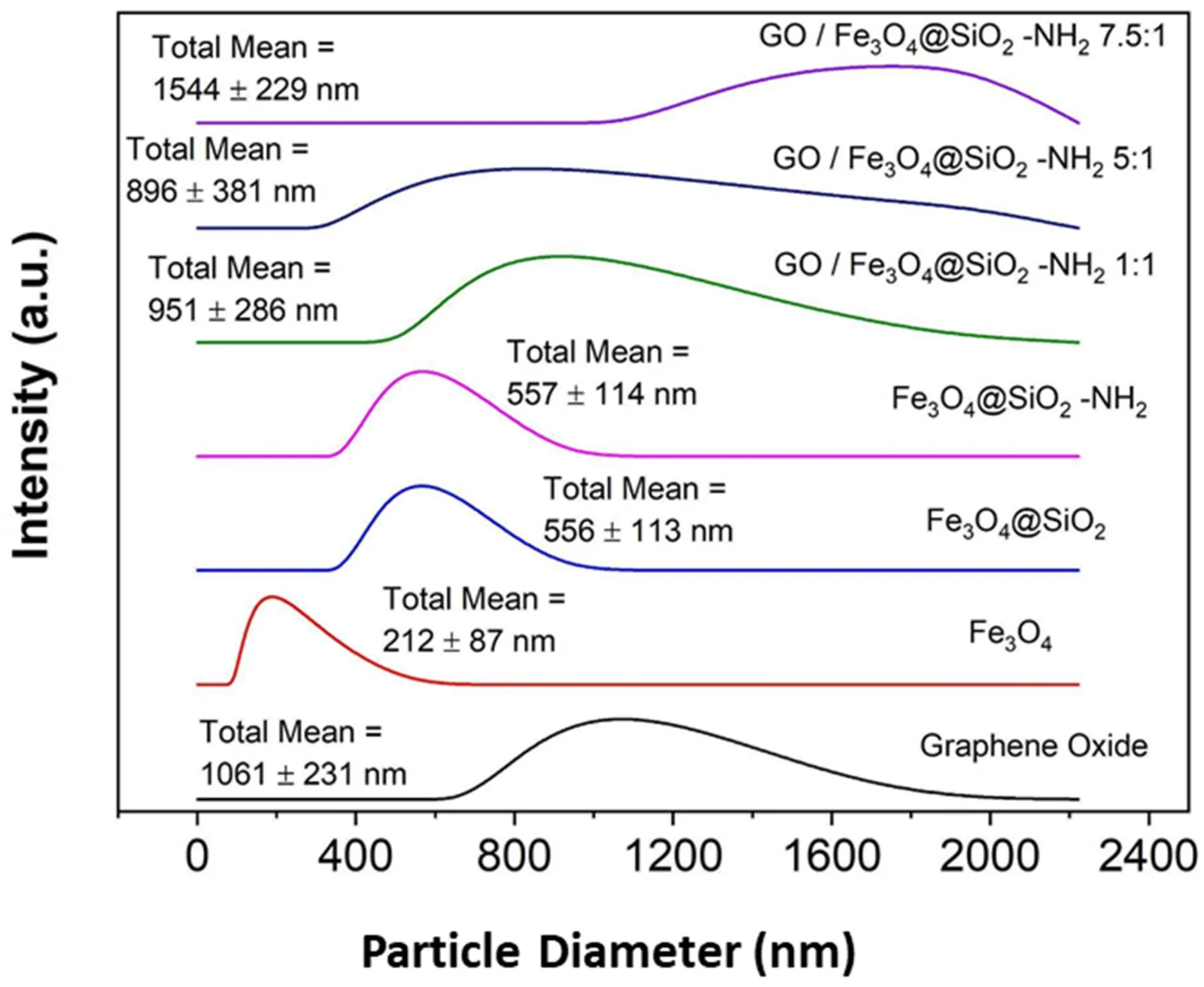
PSA characterization results for various samples.

Figure 5 shows the DLS-derived hydrodynamic diameter of the samples at different modification stages. GO exhibited an average hydrodynamic diameter of approximately 1061 nm, which can be associated with the lateral dimensions of the GO sheets and their aggregation or association in suspension. Fe_3_O_4_ nanobeads exhibited a smaller hydrodynamic diameter of approximately 212 nm, while silica coating increased the measured value to approximately 556 nm. The value remained relatively similar after amino functionalization. Following GO conjugation, hydrodynamic diameters of approximately 951 nm, 895 nm, and 1544 nm were obtained for the 1:1, 5:1, and 7.5:1 ratios, respectively. These DLS values represent the hydrodynamic dimensions of dispersed particles and particle assemblies and should not be interpreted as the primary-particle dimensions observed by TEM. In particular, the approximately 1.5 µm hydrodynamic size obtained for the 7.5:1 sample indicates the presence of relatively large assemblies for which gravitational sedimentation may occur over time. Therefore, the DLS data are used here primarily for comparative evaluation of the dispersed particle/aggregate dimensions under the stated measurement conditions rather than as evidence of long-term colloidal stability. The observed changes in hydrodynamic diameter indicate that surface functionalization influences particle association in suspension. Similar effects of surface modification on the physicochemical behavior of magnetic nanomaterials have been reported previously [27].

**Figure 6:**
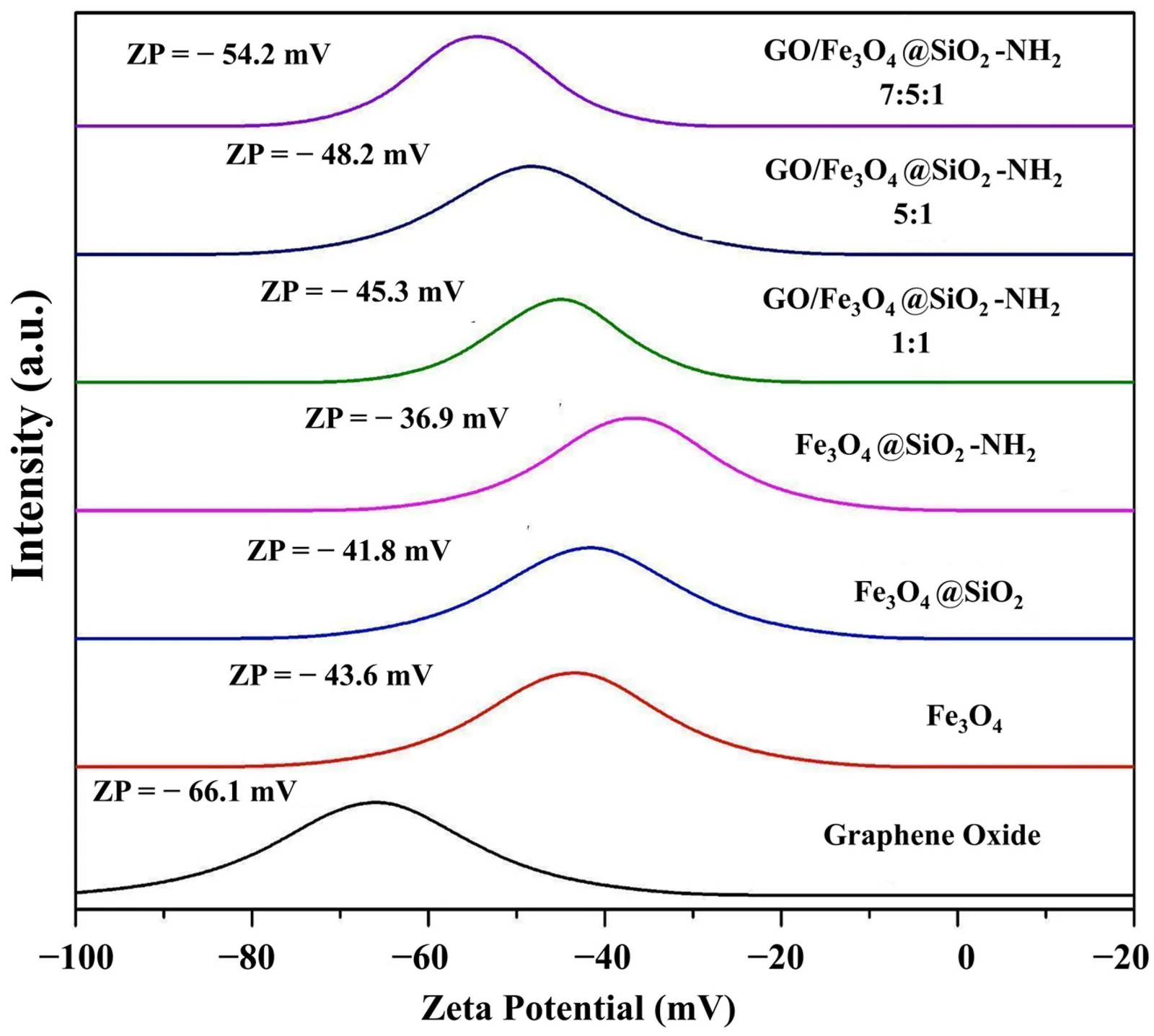
Zeta potential characterization results for various samples.

The electrokinetic properties of the nanoparticle suspensions were evaluated using zeta-potential measurements, and the results are shown in Figure 6. Due to the presence of ionizable carboxyl and hydroxyl groups, graphene oxide (GO) exhibited a strongly negative zeta potential of −66.1 mV, indicating strong electrostatic repulsion under the specified measurement conditions. The results of Priyadarsini et al. (2018) [28], highlighting the contribution of oxygen-containing functional groups to the surface charge of GO, are consistent with this observation. The zeta potential of the Fe_3_O_4_ nanobeads was −43.6 mV. After silica coating (Fe_3_O_4_@SiO_2_), the zeta potential remained close to that of Fe_3_O_4_, indicating that the silica coating produced only a limited change in the measured surface charge.

With amino functionalization (Fe_3_O_4_@SiO_2_-NH_2_), a lower-magnitude negative zeta potential of −36.9 mV was obtained, presumably due to the presence of protonated amino groups at near-neutral pH. Similar changes in surface charge following amine functionalization of Fe_3_O_4_@SiO_2_ nanoparticles have been reported previously [29]. Following GO conjugation, the zeta-potential values became progressively more negative with increasing GO ratio: −45.3 mV (1:1 ratio), −48.2 mV (5:1 ratio), and −54.2 mV (7.5:1 ratio). This trend indicates increasing electrostatic repulsion between dispersed particles with increasing GO content under the specified measurement conditions. However, considering the relatively large hydrodynamic dimensions of the GO-conjugated samples, these zeta-potential values should not be interpreted as evidence of long-term colloidal stability or resistance to gravitational sedimentation.

Considering the DLS-derived hydrodynamic diameter and zeta-potential results, the 5:1 GO-to-Fe_3_O_4_@SiO_2_-NH_2_ conjugation ratio was selected as a practical balance between particle dimensions and functional coverage for magnetic separation. This ratio provides adequate GO functionalization while maintaining a lower hydrodynamic diameter than the 7.5:1 sample. The increasingly negative zeta potential following GO conjugation reflects changes in the electrokinetic properties of the nanobeads and should not be interpreted as promoting DNA adsorption through electrostatic attraction. Since DNA is a polyanion and GO surfaces are generally negatively charged under near-neutral conditions, electrostatic repulsion between DNA and GO is expected [30,31]. Nevertheless, DNA adsorption onto GO can occur through non-electrostatic interactions, particularly π–π stacking between DNA nucleobases and graphenic domains, hydrogen bonding, and hydrophobic interactions [31,32].

**Figure A1:**
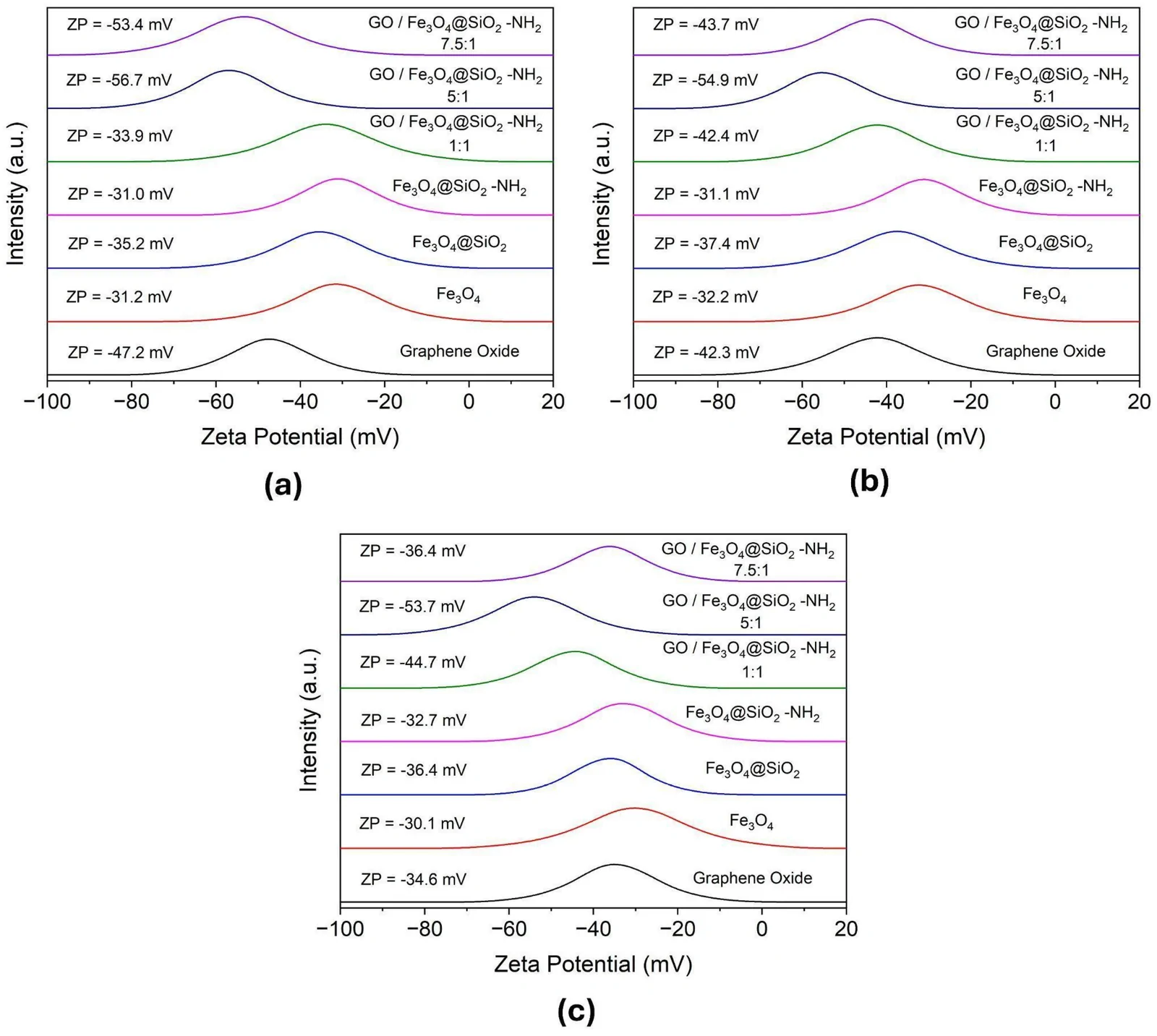
Zeta-potential distributions of various samples measured at pH 6, 7, and 8. Panels (**a**–**c**) represent measurements at pH 6, pH 7, and pH 8, respectively.

To further evaluate the electrokinetic behavior of the various samples under pH conditions relevant to DNA extraction, additional zeta-potential measurements were performed at pH 6, 7, and 8, as presented in Appendix A.2 (Figure A1). All samples maintained negative zeta-potential values across the investigated pH range, although the magnitude varied depending on the surface treatment. GO exhibited progressively less negative values with increasing pH, whereas the GO-conjugated formulations (1:1, 5:1, 7.5:1) showed distinct, non-uniform responses. These findings demonstrate that the electrokinetic properties of the nanobeads are responsive to pH while retaining their negative surface charge throughout the investigated range. The persistence of a negative surface charge from pH 6 to 8 indicates that the nanobeads preserve their characteristic electrokinetic behavior across pH conditions relevant to the DNA extraction workflow, supporting their applicability for magnetic DNA separation under varying pH environments.

**Figure 7:**
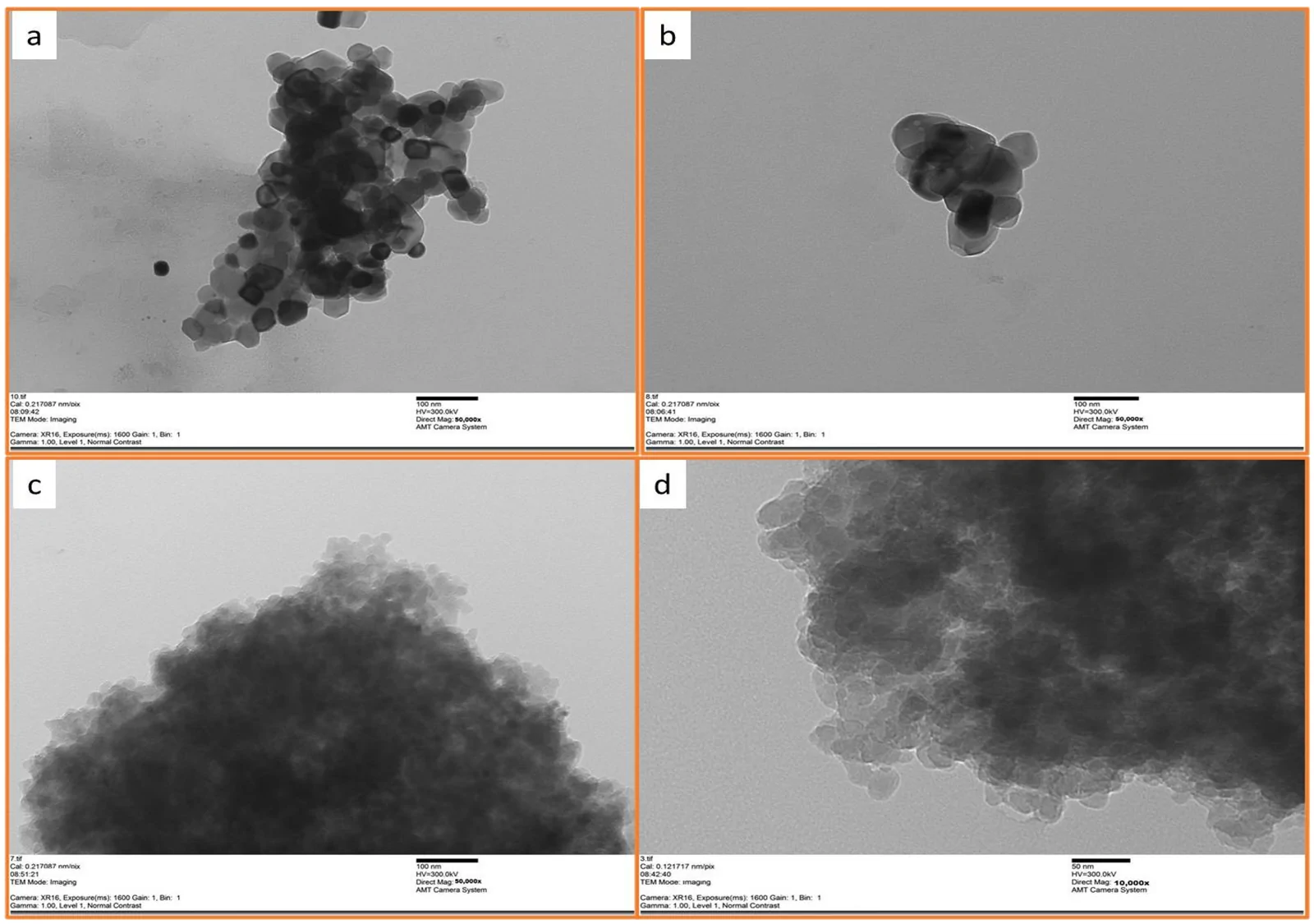

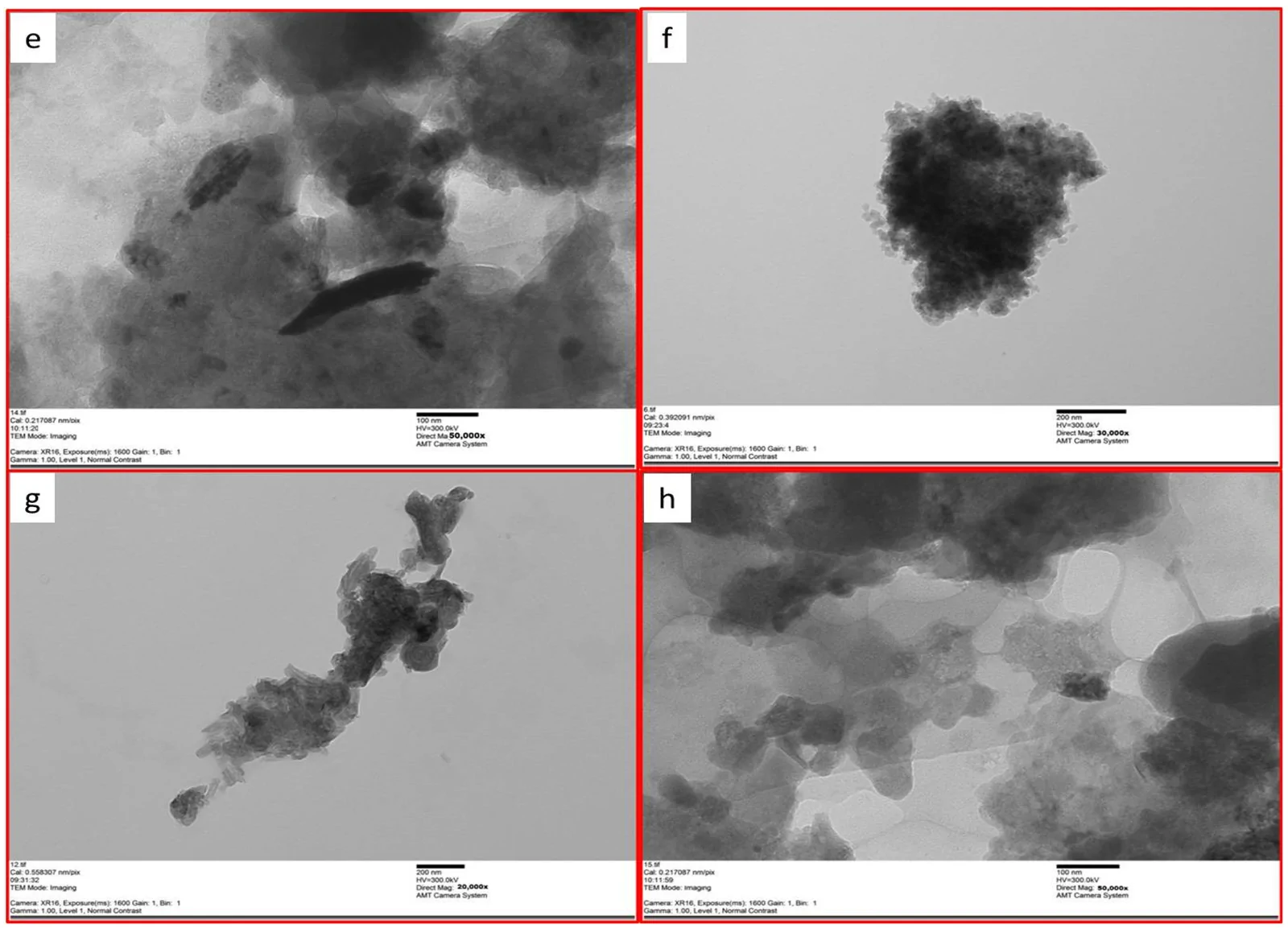
HRTEM spectrum of Fe_3_O_4_-SiO_2_ (**a**,**b**), Fe_3_O_4_-SiO_2_-NH_2_ (**c**,**d**), Fe_3_O_4_-GO (**e**,**f**), and GO (**g**,**h**).

High-resolution transmission electron microscopy (HRTEM) was used to study the microstructural evolution of the nanocomposites throughout the synthesis process (Figure 7). Figure 7 shows that the Fe_3_O_4_@SiO_2_ nanoparticles have a quasi-spherical structure with an average diameter of 63 nm. The magnetic cores are covered with a uniform silica layer (Figure 7c,d), the average diameter being reduced to that of the Fe_3_O_4_@SiO_2_ nanoparticles at 20 nm. However, the introduction of surface amine groups drives a denser, more interconnected spatial arrangement; the particles pack into compact clusters via enhanced interparticle interactions. The incorporation of graphene oxide (GO) into these functionalized particles changes the composite morphology completely. The crumpled, sheet-like (Figure 7e,f) provides a robust structural scaffold for the immobilization of nanoparticles. The strong interfacial binding leads to the localized clustering on the graphene surface, increasing the apparent average size of the assembled clusters to 89 nm and confirming the close physical contact between the two phases.

Although GO conjugation improved surface functionality, HRTEM images revealed partial aggregation of GO composites. Aggregation may reduce reproducibility of DNA extraction by limiting available binding sites. However, the optimized 5:1 ratio minimized aggregation compared to higher GO loadings, balancing stability and performance. By optimizing the synthesis conditions, Figure 7g,h shows a much better structural homogeneity. The average aggregate size decreases significantly to 55 nm, which is due to the highly efficient GO exfoliation and a much-improved dispersion of the magnetic nanoparticles. Rather than forming dense clusters, the Fe_3_O_4_@SiO_2_-NH_2_ particles anchor uniformly across the available graphene surface area. Ultimately, this microstructural progression confirms a successful stepwise assembly: from stable, well-defined magnetic core–shell structures to highly dispersed, graphene-supported hybrid platforms. The precise structural control and robust dispersion demonstrated here, particularly the architecture, provide an ideal morphological foundation for demanding applications in targeted drug delivery, environmental remediation, and advanced catalysis.

**Figure 8:**
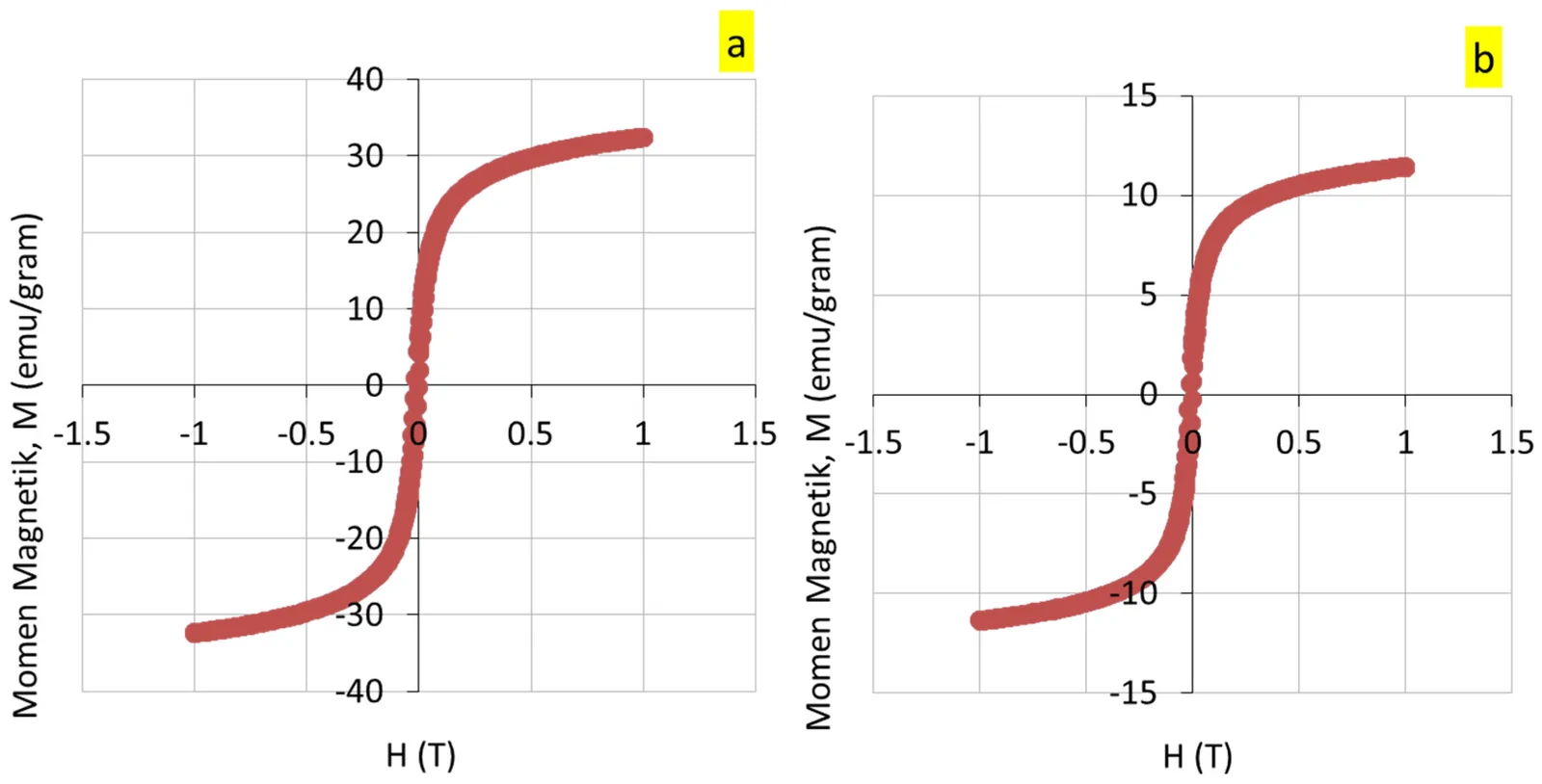
Magnetization curve of the sample Fe_3_O_4_ (**a**) and Fe_3_O_4_@SiO_2_ (**b**).

The VSM analysis of the iron oxide sample shows a typical S-shaped magnetization curve with negligible hysteresis, indicating the material possesses superparamagnetic or soft ferrimagnetic behavior (Figure 8a). The magnetization increases sharply with the applied magnetic field and gradually reaches saturation at higher field strength, confirming the strong magnetic response of the synthesized iron oxide particles. From the graph, the saturation magnetization (Ms) is approximately 32–33 emu g^−1^, which is lower than that of bulk magnetite, likely due to the nanoscale particle size, surface spin disorder, structural defects, or the presence of non-magnetic coating/capping molecules on the particle surface. The coercivity (Hc) and remanent magnetization (Mr) are nearly zero, as the curve passes through the origin with almost no loop opening, which confirms that the particles do not retain magnetization after removal of the external magnetic field. This behavior is highly desirable for applications such as magnetic separation, drug delivery, biosensing, wastewater treatment, and nucleic acid purification, where particles should respond strongly to an applied magnetic field but redisperse easily once the field is removed. However, the Fe_3_O_4_-SiO_2_ VSM curve also exhibits a characteristic S-shaped magnetization profile with almost negligible hysteresis, indicating that the material retains superparamagnetic or soft ferrimagnetic behavior even after Si addition (Figure 8b). The magnetization rises rapidly with increasing applied magnetic field and reaches a near-saturation value of about 11–12 emu g^−1^, which is significantly lower than that of undoped Fe_3_O_4_. This decrease in saturation magnetization (Ms) can be attributed to the presence of non-magnetic silica, which reduces the magnetization measured per unit mass of the composite particles, and to the incorporation of non-magnetic Si species, which dilute the magnetite Fe_3_O_4_ phase, along with possible surface spin disorder, structural defects, and reduced magnetic domain interactions caused by doping. The coercivity (Hc) and remnant magnetization (Mr) remain close to zero, as the curve passes through the origin without a noticeable hysteresis loop, confirming that the material does not retain magnetization once the external field is removed. Therefore, the VSM results confirm that although carbon doping reduces the overall magnetic strength of Fe_3_O_4_, the material still preserves its desirable superparamagnetic character.

### 3.4. Genomic DNA Extraction Performance

The performance of genomic DNA extraction using the nanodrop test produces two main indicators, namely yield and purity of the extraction results. DNA is classified as good quality if the nanodrop test results show a yield concentration of 100 ng/ul [33]. On the other hand, the A260/A280 ratio in DNA extraction is widely used for assessing the purity of nucleic acid samples; pure DNA samples normally fall within the range of 1.7 to 2.0. Ratios considerably lower than 1.6 indicate the presence of contaminants like protein and phenol, while ratios above ~2.0 suggest possible RNA contamination [34]. Fe_3_O_4_@SiO_2_ produced a DNA yield of 160.9 ng/µL with an A260/A280 ratio of 2.10. Following GO conjugation, the 1:1, 5:1, and 7.5:1 formulations produced DNA yields of 158.4, 172.2, and 350.1 ng/µL, respectively, with corresponding A260/A280 ratios of 2.02, 2.04, and 1.91. These values are based on single-run Nanodrop measurements and are therefore interpreted as preliminary performance indicators. Table 1 summarizes the present results together with several magnetic- and nanomaterial-based DNA extraction systems reported in the literature. Because the reported studies differ in sample type, extraction protocol, and reporting units, the literature data are included primarily to provide contextual comparison rather than direct quantitative ranking.

Based on these results, functionalization of Fe_3_O_4_@SiO_2_ using GO can improve the efficiency of DNA adsorption and desorption due to its striking structural and chemical properties [24]. This efficiency can occur due to interactions through hydrogen bonds and the π-π stacking mechanism, which are the main factors in the adsorption and desorption processes [42]. The π-π stacking interaction is through the aromatic rings in GO with the planar DNA nucleobases [43], while hydrogen bonds involve the interactions of GO functional groups with hydrogen donors in DNA [44]. Such a mechanism combination enhances the flexibility of the material in the process of DNA separation with strong binding and easy release compared to the non-functionalized Fe_3_O_4_@SiO_2_ surface [45]. Therefore, GO/Fe_3_O_4_@SiO_2_-NH_2_ is a promising magnetic nanobeads material for application in DNA separation.

## 4. Conclusions

GO-conjugated magnetic Fe3O_4_@SiO_2_ nanobeads have been developed and characterized with the prospect of improving DNA separation. The combination of a magnetic Fe_3_O_4_ core, stable silica shell, amino-functionalized surface, and GO layer provided favorable physicochemical properties, enhanced surface functionality, and improved colloidal stability. Various characterization techniques confirmed that GO was successfully conjugated to Fe_3_O_4_@SiO_2_-NH_2_ particles, improving the surface properties and providing many functional groups that increased the efficiency of DNA binding. The ideal GO to Fe_3_O_4_@SiO_2_-NH_2_ conjugation ratio (5:1) balanced sufficient GO coverage and controllable particle dimensions, guaranteeing effective magnetic separation. The zeta-potential measurements showed increasingly negative values with increasing GO content, indicating enhanced electrostatic repulsion under the specified measurement conditions. Nevertheless, because the GO-conjugated samples exhibited relatively large hydrodynamic dimensions, including values approaching 1.5 µm, the zeta-potential results are not interpreted as evidence of long-term colloidal stability or resistance to gravitational sedimentation. This result is consistent with the results of zeta potential. It is concluded that GO-conjugated magnetic Fe_3_O_4_@SiO_2_ nanobeads are an excellent material for complex DNA separation techniques.

## Figures and Tables

**Table 1:** Genomic DNA yields and purity using different samples.

No.	Materials	DNA Yield (ng/µL)	Purity (A260/A280 Ratio)	References
1.	γ-Fe_2_O_3_/PEDOT	8.6 μg	1.97	[35]
2.	Silica-Coated Magnetic Nanoparticles (Fe_3_O_4_/SiO_2_)	116.63 µg	1.70–1.95	[36]
3.	Polyethylenimine-Modified Magnetic Gold (Fe_3_O_4_/Au-PEI)	43.1 µg/10 mL	~1.81	[37]
4.	Ferrite-Based Nanoparticles (MgFe_2_O_4_, MnFe_2_O_4_)	696 µg/mL	1.60–2.00	[38]
5.	Titanium Dioxide Nanoparticles (TiO_2_ NPs)	56.92% (gDNA)/66.31% (pDNA) recovery rate	1.75–1.90	[39]
6.	Guanidinium Ionic Liquid-Magnetic Chitosan Graphene Oxide (GIL-MCGO)	19.6 μg	1.87–1.85	[40]
7.	Multi-Walled Carbon Nanotubes Phenol Composite (MWCNT-g-PCA-Phenol)	944–785 µg g^−1^	1.80–1.90	[41]
8.	Fe_3_O_4_@SiO_2_	160.9	2.10	Present work
	GO/Fe_3_O_4_@SiO_2_-NH_2_ 1:1	158.4	2.02	
	GO/Fe_3_O_4_@SiO_2_-NH_2_ 5:1	172.2	2.04	
	GO/Fe_3_O_4_@SiO_2_-NH_2_ 7.5:1	350.1	1.91	

## Data Availability

The original contributions presented in this study are included in the article. Further inquiries can be directed to the corresponding author.

## Appendix A

### Appendix A.1

**Table A1:** Elemental composition of Fe_3_O_4_ and Fe_3_O_4_@SiO_2_ nanoparticles determined by X-ray fluorescence (XRF) analysis.

No.	Element	Fe_3_O_4_ (Mass%)	Fe_3_O_4_@SiO_2_ (Mass%)
1	Fe	99.8	65.9
2	Si	0.241	34.1
3	Mg	NR	NR
4	Zr	NR	NR

Note: NR = not reported. The selected XRF reports provide quantitative results for Fe and Si only. Mg and Zr were not included in these reports; therefore, their absence cannot be confirmed. All reported mass percentages are presented as provided by the instrument, without additional normalization.
